# Chromosome Dynamics in Bacteria: Triggering Replication at the Opposite Location and Segregation in the Opposite Direction

**DOI:** 10.1128/mBio.01002-19

**Published:** 2019-07-30

**Authors:** Ady B. Meléndez, Inoka P. Menikpurage, Paola E. Mera

**Affiliations:** a Department of Chemistry and Biochemistry, New Mexico State University, Las Cruces, New Mexico, USA; Vanderbilt University

**Keywords:** *Caulobacter crescentus*, DnaA, ParA, centromere, chromosome replication, chromosome segregation

## Abstract

Maintaining the integrity of the genome is essential to cell survival. In the bacterium Caulobacter crescentus, the single circular chromosome exhibits a specific orientation in the cell, with the replication origin (*ori*) residing at the pole of the cell bearing a stalk. Upon initiation of replication, the duplicated centromere-like region *parS* and *ori* move rapidly to the opposite pole where *parS* is captured by a microdomain hosting a unique set of proteins that contribute to the identity of progeny cells. Many questions remain as to how this organization is maintained. In this study, we constructed strains of *Caulobacter* in which *ori* and the *parS* centromere can be induced to move to the opposite cell pole in the absence of chromosome replication, allowing us to ask whether once these chromosomal foci were positioned at the wrong pole, replication initiation and chromosome segregation can proceed in the opposite orientation. Our data reveal that DnaA can initiate replication and ParA can orchestrate segregation from either cell pole. The cell reconstructs the organization of its ParA gradient in the opposite orientation to segregate one replicated centromere from the new pole toward the stalked pole (i.e., opposite direction), while displaying no detectable viability defects. Thus, the unique polar microdomains exhibit remarkable flexibility in serving as a platform for directional chromosome segregation along the long axis of the cell.

## Abstract (precis)

Bacteria can accomplish surprising levels of organization in the absence of membrane organelles by constructing subcellular asymmetric protein gradients. These gradients are composed of regulators that can either trigger or inhibit cell cycle events from distinct cell poles. In Caulobacter crescentus, the onset of chromosome replication and segregation from the stalked pole are regulated by asymmetric protein gradients. We show that the activators of chromosome replication and segregation are not restricted to the stalked pole and that their organization and directionality can be flipped in orientation. Our results also indicate that the subcellular location of key chromosomal loci play important roles in the establishment of the asymmetric organization of cell cycle regulators.

## INTRODUCTION

Triggering the onset of chromosome replication under the right conditions is central to cell survival and proliferation. In bacteria, DnaA is the highly conserved initiator of chromosome replication that opens the origin of replication (*ori*) by forming a helical right-handed polymeric structure (1–5). Once DnaA opens the double-stranded chromosome, the replication machinery assembles at *ori* and initiates chromosome replication bidirectionally. The subcellular location where DnaA initiates chromosome replication is established by the position of *ori* inside the cell. Depending on the bacterial species, the subcellular location of *ori* varies significantly. For instance, *ori* in Caulobacter crescentus (referred to hereafter as *Caulobacter*) is found at one pole (the stalked pole), whereas in Escherichia coli and Bacillus subtilis, *ori* is found near mid-cell. Within a single species, the subcellular location of *ori* is strictly retained at the same position in nondividing cells and reestablished soon after chromosome replication and segregation initiate in actively dividing cells.

**FIG 1:**
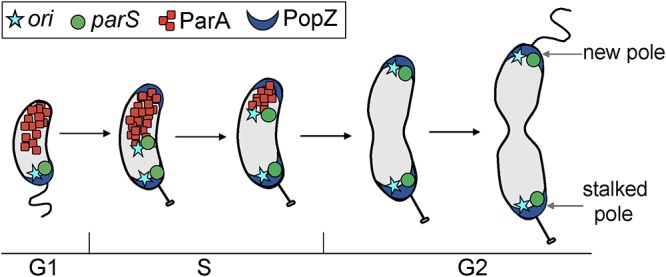
Depiction of cell cycle-dependent dynamics of Caulobacter crescentus. Localization of two chromosomal foci (origin of replication [*ori*] and centromere [*parS*]) and two proteins involved in chromosome segregation (ParA and PopZ) over the course of a normal cell cycle. Nondividing cells have *ori* (cyan) and *parS* (green) localized near the stalked pole. PopZ anchors the centromere region *parS* and the *parS*-binding protein ParB complex at the stalked pole. Once replication initiates, two foci of *ori* and two foci of *parS* are observed. Upon the onset of chromosome replication and segregation, a second PopZ foci appears at the new pole. The new duplicated *ori* and *parS* are the first regions to move in a ParA-dependent manner to the new pole.

In nondividing *Caulobacter*, *ori* is retained near the stalked pole by the interaction between the anchoring protein PopZ and the ParB partition protein bound to the centromere-like chromosomal *parS* (referred to hereafter also as the centromere) (6–8). DnaA initiates replication at *ori* near the stalked pole (Fig. 1). Once chromosome replication initiates at *ori*, the replication fork must pass through the centromere chromosomal region (*parS*, 8 kb away from *ori*) for chromosome segregation to initiate (9). One of the two newly replicated ParB-coated centromeres is segregated to the opposite pole (also referred to as the new pole) by direct interactions between ParB and the ATPase ParA (9–14). It has been proposed that ParA forms a stable gradient with concentrations gradually decreasing from the new pole to the stalked pole, which are critical for establishing segregation directionality (10–12, 15).

In this report, we asked whether the onset of replication and segregation are restricted to the intrinsic localization of *ori* and the centromere, which in *Caulobacter* are near the stalked pole. We genetically engineered a *Caulobacter* strain where movement of *ori* and the *parS* centromere can be triggered in the absence of replication initiation (16). Once these chromosomal foci were translocated to the opposite cell pole, we tested for competency of replication initiation and chromosome segregation. Our data demonstrate that DnaA and ParA are able to initiate chromosome replication and segregation irrespective of the subcellular localization of *ori* and the centromere. The organization of the ParA gradient flips in orientation once the unreplicated centromere is relocalized to the new pole, revealing the robustness and flexibility of the orientation of chromosome replication and segregation.

## RESULTS

### Subphysiological levels of DnaA result in translocation of *ori* away from the stalked pole.

**FIG 2:**
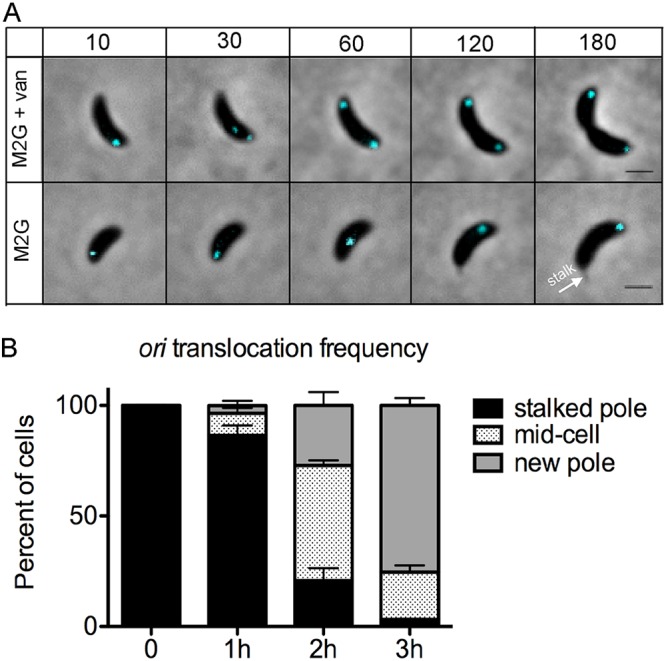
Translocation of *ori* in the absence of chromosome replication. (A) Time lapse of indicator strain [PM500; *parS*(pMT1) *vanA*::*dnaA* Δ*dnaA xylX*::*cfp*-*parB*(pMT1)] with fluorescent tag near *ori* (∼1 kb) with *dnaA* expression regulated by the vanillate promoter. Cells grown in M2G with vanillate were supplemented with xylose (0.3%) for 1 h and synchronized. Swarmer cells were spotted on 1% agarose M2G pads in the presence of vanillate (250 μM) (top row) or absence of vanillate (bottom row). Cells were imaged with phase-contrast and CFP-mediated fluorescence microscopy every 30 min. The time in minutes is shown above the images. The white arrow indicates the location of the stalk. Bars = 1 μm. (B) Percentage of cells with translocated *ori* to the middle or new pole over a 3-h span of DnaA depletion. Values are means plus standard deviation (SD) (error bars) percentages from three independent experiments. The average number of cells per replicate was 250.

In *Caulobacter*, the centromere is the first chromosomal locus to segregate away from the stalked pole (9). Previous analyses of cells expressing subphysiological levels of DnaA (insufficient to initiate replication) revealed a DnaA-dependent and replication-independent segregation of the centromere (16). Subphysiological levels of DnaA cause the unreplicated centromere to move in a ParA-dependent manner from the stalked pole to the new pole (16). We asked whether subphysiological levels of DnaA could also trigger the movement of *ori* independently of replication. We tracked the localization of *ori* by constructing a strain with a fluorescent tag to be inserted near *ori* using the Yersinia pestis *parS*(pMT1) chromosomal sequence and its corresponding gene encoding ParB(pMT1) (17). This strain is called PM500 [*xylX*::*cfp*-*parB*(pMT1), *parS*(pMT1) at nucleotide 1108, *dnaA*::Ω, *vanA*::*dnaA*; details on strain construction found in Materials and Methods]. Using growth curves and CFU, we showed that this strain, PM500 with the *parS*(pMT1) insertion near *ori* and expression of *cfp*-*parB*(pMT1), exhibits no significant effect on doubling time and viability compared to the wild type (see Fig. S1 in the supplemental material). To reach subphysiological levels of DnaA in the cell, we used the same vanillate promoter that was used to regulate *dnaA* expression when tracking centromere movement (16). Our results show that subphysiological levels of DnaA trigger the translocation of *ori* away from the stalked pole independently of chromosome replication (Fig. 2A; see also Movie S1 in the supplemental material). As the time of DnaA depletion increased, cells displayed unreplicated *ori* foci progressively moving toward the new pole (Fig. 2B). Within 3 h of DnaA depletion, ∼75% of cells exhibited *ori* localized at the new pole. These results resemble the replication-independent movement of the centromere, suggesting that *ori* and the centromere translocate in a DnaA-dependent manner likely due to their close proximity on the chromosome (16).

10.1128/mBio.01002-19.1 FIG S1 Construction of Y. pestis *parS*/*cfp*-*parB*(pMT1) system into *Caulobacter* near *ori* does not alter cell viability. (A) Exponential state growth curves (*n* = 3) of *Caulobacter* WT (PM1; NA1000, wild-type), *parS*(pMT1) (PM433; cells with Y. pestis *parS* sequence inserted near *ori*), and *parS*/*cfp*-*parB*(pMT1) (PM438; PM433 with CFP-ParB from Y. pestis expressed from xylose-inducible promoter). Cells were grown in minimal media (M2G). The cultures (2 ml) were set to an OD_600_ of ∼0.2, and growth was monitored by measuring the absorbance at 600 nm every hour. Expression of *cfp*-*parB*(pMT1) was induced by adding xylose (0.3%). (B) The colony-forming unit (CFU) assay of wild-type (WT) *Caulobacter* (PM1), *parS*(pMT1) (PM433), and *parS*/*cfp*-*parB*(pMT1) (PM438) on PYE plates in the presence or absence of xylose (0.3%). CFU assays were conducted as described in Materials and Methods. Cultures were grown at 28°C, and the CFU plates were incubated for 2 days before imaging. Data shown are representative of three independent replicates. Download FIG S1, TIF file, 1.7 MB. Copyright © 2019 Meléndez et al. 2019 Meléndez et al. This content is distributed under the terms of the Creative Commons Attribution 4.0 International license.

10.1128/mBio.01002-19.6 MOVIE S1 Time-lapse movie to show PM500 cells translocating *ori* in the absence of chromosome replication. Scale bar, 2 μm. Download Movie S1, MOV file, 2.7 MB. Copyright © 2019 Meléndez et al. 2019 Meléndez et al. This content is distributed under the terms of the Creative Commons Attribution 4.0 International license.

### DnaA’s ability to initiate replication is not restricted to the stalked pole.

**FIG 3:**
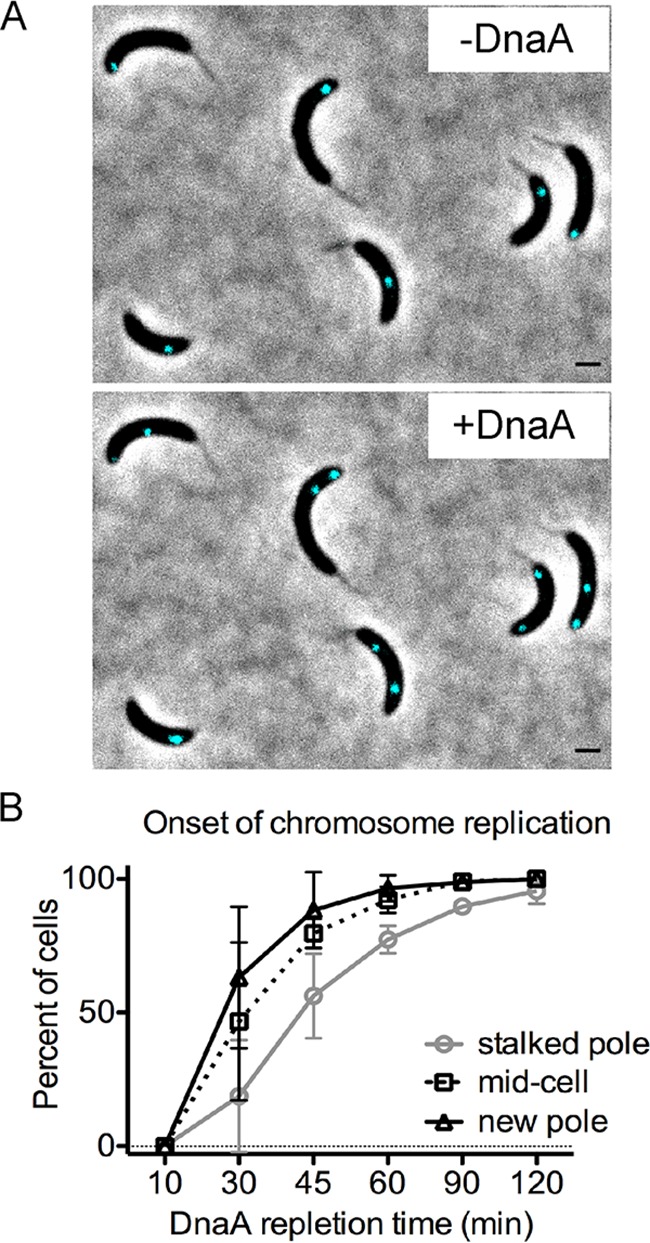
The onset of chromosome replication is not limited to the stalked pole. (A) Cells with fluorescent tag near *ori* [PM500; *parS*(pMT1) near *ori*, *vanA*::*dnaA*, D*dnaA*, p*xylX*::*cfp*-*parB*(pMT1)] were grown in M2G with vanillate and supplemented with xylose (0.3%) for 1 h prior to synchronization. DnaA was depleted by growing the cells in liquid M2G medium without vanillate. (Top) At 3 h of DnaA depletion, cells displayed unreplicated *ori* foci translocated to the opposite pole, middle, or at the stalked pole. After the depletion period, DnaA expression was induced by supplementation of vanillate (250 μM). (Bottom) Within 30 min of DnaA repletion, cells were able to initiate chromosome replication, as evidenced by two *ori* (cyan) foci. Bars = 1 μm. (B) Onset of chromosome replication starting from the stalked pole, mid-cell, or new pole of PM500. The plot represents the mean ± SD percentage of cells with two *ori* foci from three independent fluorescence microscopy time-lapses. The average number of cells per replicate was 140. Analyses of two-way analysis of variance (ANOVA) between the frequencies of replication at the stalk pole versus new pole are statistically different at the 30-min and 45-min time points (*P* < 0.001; *P* < 0.05).

In *Caulobacter*, DnaA initiates replication only once per cell cycle and only in stalked cells, which have their *ori* localized at the stalked pole (Fig. 1). To determine whether DnaA can initiate replication at the opposite cell pole, we analyzed cells that had undergone replication-independent translocation of *ori*. To track replication initiation, we induced *dnaA* from a vanillate promoter in cells with *ori* localized to the opposite cell pole and followed the appearance of two newly replicated *ori* foci (Fig. 3A; Movie S2). We quantified the frequencies of replication initiation based on the initial location of *ori* prior to induction of *dnaA* expression (Fig. 3B). Within 30 min of vanillate supplementation, ∼63% of cells with *ori* localized at the new pole had initiated chromosome replication as evidenced by two clearly separated *ori* foci. Notably, chromosome replication initiated slower in the subpopulation of cells that retained *ori* at the stalked pole: ∼19% of cells displayed two *ori* foci after 30 min of vanillate supplementation. Our data revealed that DnaA’s activity as a replication initiator is not restricted to the stalked pole.

10.1128/mBio.01002-19.7 MOVIE S2 Time-lapse movie to show chromosome replication after DnaA induction in PM500 cells that had the unreplicated *ori* translocated to different subcellular locations. Scale bar, 2 μm. Download Movie S2, MOV file, 3.2 MB. Copyright © 2019 Meléndez et al. 2019 Meléndez et al. This content is distributed under the terms of the Creative Commons Attribution 4.0 International license.

### Centromeres are effectively segregated in the opposite direction.

On the basis of our results that DnaA is able to initiate replication from the new pole (Fig. 3), we then asked about chromosome segregation. Can the partitioning system ParABS initiate segregation of the centromere from the new pole toward the stalked pole, which in this case would be in the opposite direction? To test this, we used a *Caulobacter* strain in which the native *parB* gene was replaced with the fusion gene encoding cyan fluorescent protein (CFP)-ParB and in which the only copy of *dnaA* was regulated under the vanillate promoter (PM109) (16). In *Caulobacter*, the partitioning protein ParB binds directly to the centromere (9, 18). Thus, we can track centromere movement by using cells expressing a functional fusion protein CFP-ParB. When the vanillate inducer is removed from the growth medium of strain PM109, cells are exposed to subphysiological levels of DnaA insufficient to initiate chromosome replication but sufficient to trigger movement of the unreplicated centromere (16). Fluorescent imaging of PM109 cells depleted of DnaA (incubated without vanillate for 3 h) revealed the localization of a single CFP-ParB focus with the following distribution: ∼37% at or near the new pole, ∼54% at or near the stalked pole, and ∼10% at/around mid-cell (Fig. S2), consistent with previous analyses (16). One potential explanation for the difference in frequencies of replication-independent *parS*-*ori* translocation between strains PM109 and PM500 (∼37% versus ∼75%) may have to do with expression of the Y. pestis *parB*(pMT1). Although we did not observe defects on doubling times and/or viability from expression of *parB*(pMT1) in *Caulobacter*, our translocation frequency data suggest that Y. pestis ParB may influence the activity of *Caulobacter*’s ParA in chromosome segregation. From here on, we use only *Caulobacter* strains with its native *parB* fluorescently tagged.

10.1128/mBio.01002-19.2 FIG S2 Frequencies of DnaA-dependent replication-independent centromere translocation. Localization of CFP-ParB foci (centromere) at or near the stalked pole, mid-cell, and at or near the new pole of *Caulobacter* cells were calculated in DnaA-depleted cells (Δ*vanA parB*::*cfp-parB dnaA*::Ω *vanA*::*dnaA*; PM109) for 3 h. Data represent the means ± SD for three independent replicates. The average number of cells per replicate was 170. Download FIG S2, TIF file, 0.9 MB. Copyright © 2019 Meléndez et al. 2019 Meléndez et al. This content is distributed under the terms of the Creative Commons Attribution 4.0 International license.

**FIG 4:**
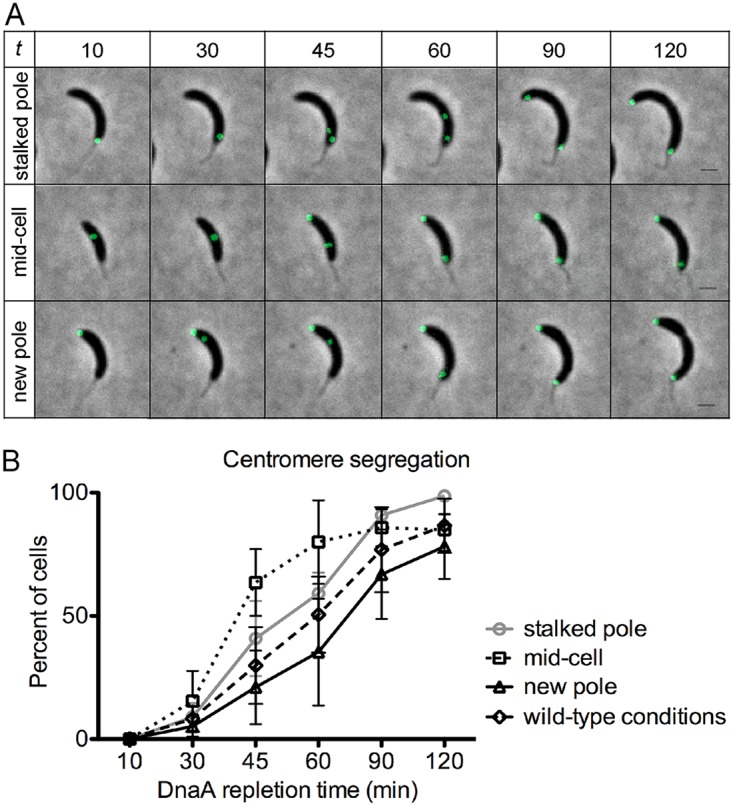
Translocated centromeres effectively segregate in the opposite direction. Cells imaged were synchronized prior to DnaA depletion in M2G medium (2 ml, OD_600_ of ∼0.1) for 3 h, and vanillate (250 μM) was added (time zero) to induce the expression of DnaA. Cells (2 μl) were spotted on 1% agarose pads supplemented with vanillate (250 μM). (A) Time-lapse microscopy of centromere segregation (green foci represent CFP-ParB/*parS*) starting from the stalked pole, mid-cell, or new pole of PM109 (*parB*::*cfp*-*parB*, *dnaA*::W, *vanA*::*dnaA*). *t* is DnaA repletion time (in minutes). Bars = 1 μm. (B) Centromere segregation. Segregation of centromeres of PM109 was quantified from three independent fluorescence microscopy time-lapse experiments. The average number of cells per replicate was 170. The plot also includes comparison of centromere segregation under wild-type conditions (dashed line). The mean ± SD percentages of cells with centromere segregation to the cell poles based on initial localization of CFP-ParB/*parS* are shown. Statistical analyses of two-way ANOVA between the frequencies of segregation at mid-cell and new pole are significantly different at the 45-min and 60-min time points (*P* < 0.01) and the frequencies of segregation at/near stalked pole and mid-cell at 45 min (*P* < 0.05).

To determine the ability of ParABS to trigger segregation of the centromere in the opposite direction (from the new pole toward the stalked pole), we tracked the movement of CFP-ParB in PM109 cells with translocated unreplicated centromeres subsequent to the addition of vanillate (Fig. 4). Two clearly separated centromeres were observed soon after *dnaA* expression was reestablished, irrespective of the initial localization of centromere (Fig. 4A; Movie S3). Upon induction of *dnaA* expression, we observed a similar pattern in the rates of replication initiation when tracking the number of *parS* centromeres. The appearance of two CFP-ParB foci occurred slightly sooner in cells with a centromere at the new pole compared to cells with a centromere at the stalked pole (Fig. S3).

10.1128/mBio.01002-19.3 FIG S3 Frequencies of chromosome replication initiation based on centromere localization. The percentages of *Caulobacter* cells (Δ*vanA parB*::*cfp-parB dnaA*::Ω *vanA*::*dnaA*; PM109) with two CFP-ParB foci based on the initial localization of *parS* (8 kb away from *ori*) are plotted. DnaA was depleted for three hours in swarmer cells isolated from mini-synchrony. Vanillate (250 μM) was added to the media (time 0 min), and the cells (2 μl) were spotted immediately on an agarose pad (1% in M2G medium) supplemented with vanillate (250 μM). Time-lapse images were obtained using phase-contrast microscopy at the given time intervals, and the appearance of two replicated centromeres were monitored. Data represent the means ± SDs for three independent replicates. The average number of cells per replicate was 170. Comparison of the two-way ANOVA between the frequencies of replication initiation at or near the stalked pole and mid-cell or the new pole were significantly different at 30-min and 45-min time points (for stalked pole and mid-cell, *, *P* < 0.05; for stalked pole and new pole, ***, *P* < 0.001 at 30 min; *, *P* < 0.05 at 45 min). Download FIG S3, TIF file, 1.1 MB. Copyright © 2019 Meléndez et al. 2019 Meléndez et al. This content is distributed under the terms of the Creative Commons Attribution 4.0 International license.

10.1128/mBio.01002-19.8 MOVIE S3 Time-lapse movie to show chromosome replication after DnaA induction in PM109 cells that had the unreplicated centromere *parS* translocated to different subcellular locations. Download Movie S3, MOV file, 4.7 MB. Copyright © 2019 Meléndez et al. 2019 Meléndez et al. This content is distributed under the terms of the Creative Commons Attribution 4.0 International license.

Notably, cells with two CFP-ParB foci were able to segregate their centromeres to the cell poles, irrespective of the initial localization of centromere prior to *dnaA* induction (Fig. 4A). Quantification of these data revealed that the rate at which centromeres were segregated to the cell poles were significantly faster in cells with centromeres that departed from the mid-cell (Fig. 4B). These results can potentially be explained based on the shorter distance that the replicated centromeres had to travel from mid-cell to reach the cell poles. The rates of centromere segregation that initiated from either the stalked pole or the new pole were not statistically different. These data suggest that the partitioning protein ParA was able to quickly rearrange its gradient in order to segregate centromeres from mid-cell or from the new pole with no significant delays.

### Active ParA is required for centromere segregation in the opposite direction.

**FIG 5:**
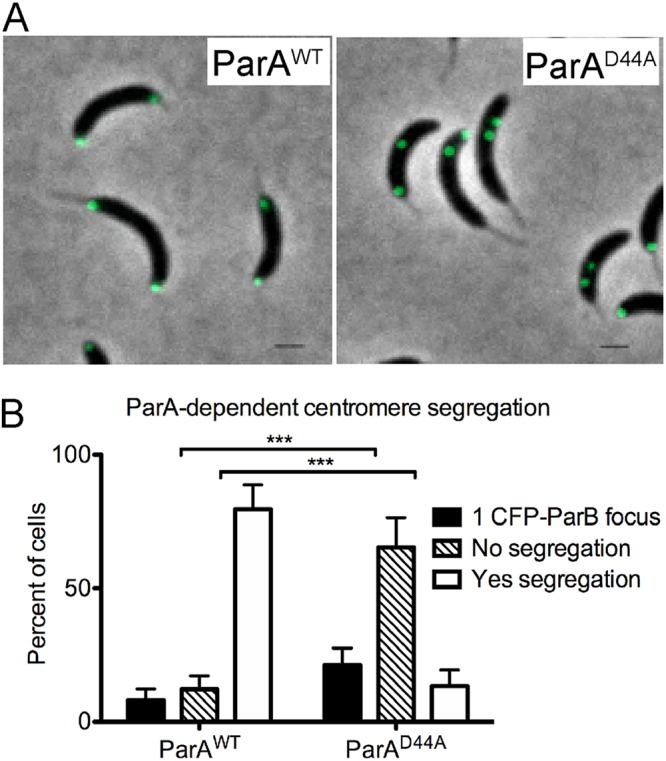
Centromere segregation in the opposite direction requires active ParA. Cells with background *parB*::*cfp*-*parB*, *dnaA*::Ω, *vanA*::*dnaA* with either wild-type ParA (ParA^WT^) (PM109) or ParA^D44A^ (PM121) were grown in the absence of vanillate to allow for centromere translocation. After 3 h of DnaA depletion, cultures were supplemented with vanillate (250 μM) to express DnaA and with xylose (0.3%) to induce the expression of ParA^D44A^ variant protein. Cells were imaged by spotting 2 μl of cells on 1% agarose pads. (A) Phase-contrast fluorescence micrographs of PM109 expressing ParA^WT^ (average cells per replicate = 250) and PM121 expressing ATP hydrolysis variant ParA^D44A^ (average cells per replicate = 225). Bars = 1 μm. (b) Frequencies of centromere segregation were quantified based on localization of CFP-ParB (green foci). The data represent analyses of three independent experiments. Bar graph illustrates the mean ± SD values. Statistical analysis: two-way ANOVA, ***, *P* < 0.001.

*Caulobacter* cells expressing ParA variants unable to hydrolyze ATP cannot segregate their centromere to the cell poles (9, 10, 14). To determine whether centromere segregation observed from the new pole or mid-cell is ParA dependent, we tracked CFP-ParB localization in a merodiploid strain that carries the wild-type allele of *parA* at the native locus and a dominant-negative mutant *parA* allele unable to hydrolyze ATP expressed from a xylose-inducible promoter. This dominant-negative allele contains a missense mutation in the ATPase domain (ParA^D44A^) of ParA that inhibits chromosome segregation (19). To test segregation in the opposite direction, we first allowed cells to translocate their unreplicated centromeres by growing them in growth media devoid of vanillate. The growth media were then supplemented with xylose and vanillate so that replication initiation was induced in the presence of the dominant-negative ParA^D44A^. Our data revealed that >80% of cells expressing wild-type ParA were able to segregate the centromeres to the poles, as evidenced by one CFP-ParB focus at each pole (Fig. 5A). However, cells expressing the dominant-negative ParA^D44A^ after replication initiated at the opposite cell pole failed to segregate their replicated centromeres as evidenced by two CFP-ParB near each other (Fig. 5A and B). These data strongly suggest that the segregation of the centromere in the opposite direction, from the new pole to the stalked pole, requires an active chromosome segregation machinery.

### Relocalization of the centromere locus triggers rearrangement of the ParA gradient.

**FIG 6:**
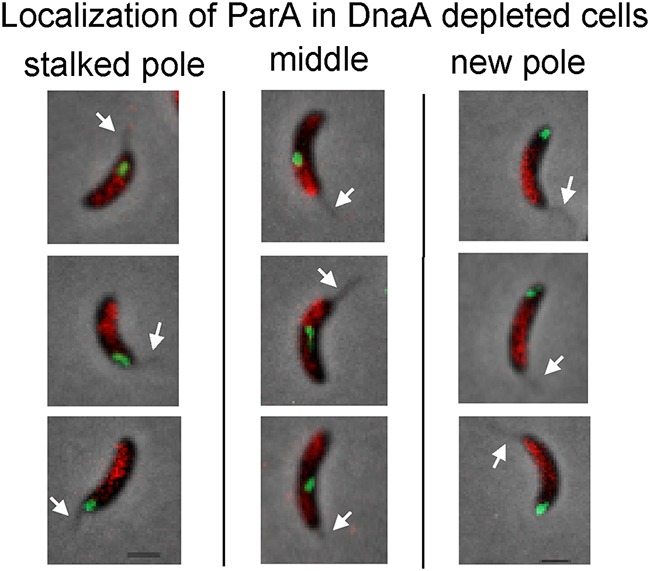
Localization of ParA depends on the subcellular localization of *parS*-ParB. Translocation of centromere to the new pole in DnaA-depleted cells results in flipped ParA-mCherry gradient (red). Green foci represent *parS*-CFP-ParB (centromeres) localized at the stalked pole, mid-cell, or at the new pole. White arrows indicate locations of the stalks. PM503 cells (*parB*::*cfp*-*parB*, *dnaA*::Ω, *vanA*::*dnaA xylX*::*parA*-mCherry) were synchronized and depleted of DnaA for 3 h. The culture was also supplemented with xylose (0.1%) during the time of DnaA depletion. After DnaA depletion, 2 μl of cells was mounted on 1% agarose pad and imaged using phase-contrast fluorescence microscopy. Micrographs were spliced to show cells with a single *parS*-CFP-ParB focus grouped based on the subcellular location of that focus (stalked pole, mid-cell, new pole). Bars = 1 μm.

In *Caulobacter*, ParA forms a visible gradient with concentrations gradually decreasing from the new pole to the stalked pole (10–12, 20) (Fig. 1). Our observation that the centromere could be segregated in the opposite direction suggested that cells with centromeres at the new pole rearrange the gradient of ParA. To determine whether ParA could change the orientation of its gradient, we assessed the localization patterns of ParA using the background of a *parA* merodiploid strain that contained the wild-type allele of *parA* at the native locus and a fluorescently tagged *parA* (ParA-mCherry) under the inducible promoter for xylose (19). We found that the simultaneous overexpression of ParA (native ParA plus ParA-mCherry) and DnaA depletion resulted in chromosome replication initiation in approximately 45% of cells, suggestive of the coregulation of DnaA and ParA observed in B. subtilis (21). However, all cells that retained their unreplicated centromere at the stalked pole displayed the ParA-mCherry gradient that resembles the gradient of wild-type cells (Fig. 6). Notably, cells with unreplicated centromeres that had translocated to the new pole displayed a flipped pattern of ParA-mCherry (high levels at the stalked pole and low levels at the new pole). When the centromere was localized at mid-cell, ParA-mCherry displayed what appears to be two separate gradients with high levels starting from both cell poles (Fig. 6). Thus, our data suggest that the subcellular localization of *parS*-*ori* plays a role in dictating the organization of ParA.

### PopZ subcellular localization patterns based on *parS* localization.

**FIG 7:**
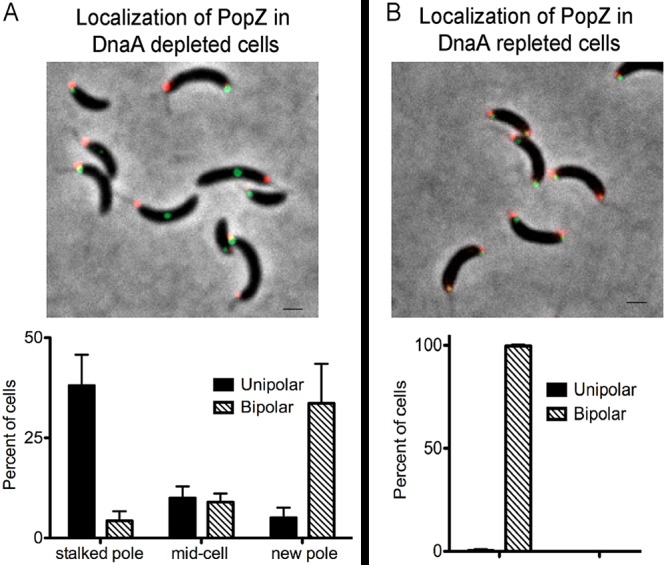
Localization of PopZ based on the subcellular organization of *parS*-ParB. (A and B) Localization of PopZ (red) in DnaA-depleted cells (A) and in 1 h DnaA replete cells (B). Green foci represent CFP-ParB (centromeres). Bars = 1 μm. The graphs display quantification of localization of PopZ in cells depleted of DnaA (A) and DnaA replete cells (B). PM247 (*parB*::*cfp*-*parB*, *dnaA*::Ω, *vanA*::*dnaA xylX*::mCherry-*popZ*) were grown in the absence of vanillate (DnaA depletion) for 3 h and then supplemented with vanillate (DnaA repletion) for 1 h. Cultures were supplemented with xylose (0.1%) to induce the expression of mCherry-PopZ for 1 h prior to isolation of swarmer cells. Phase-contrast fluorescent micrographs were obtained just before and after 1 h of the addition of vanillate. The data represent three independent experiments. The average number of cells per replicate was 200. The bar graphs show the mean plus SD values.

The swarmer exhibits a single PopZ focus at the pole bearing the flagellum. As the swarmer cell differentiates into a stalked cell and initiates DNA replication, a second PopZ focus is established at the opposite pole (new pole) (6–8). The directionality of ParA’s function in centromere segregation from the stalked pole to the new pole has been proposed to be influenced by the localization of the anchoring protein PopZ (19, 22). To determine whether the onset of chromosome replication/segregation from the new cell pole altered PopZ localization dynamics, we tracked the localization of PopZ by using cells expressing a functional fusion protein mCherry-PopZ. In our control experiment with cells grown in the presence of vanillate (the *dnaA* inducer), mCherry-PopZ exhibited foci at each pole upon the onset of chromosome replication and segregation (6–8, 16). In cells with translocated unreplicated centromeres, mCherry-PopZ localization was dependent on the localization of CFP-ParB bound to the *parS* centromere (Fig. 7A). About 90% of cells with CFP-ParB at the stalked pole displayed a single mCherry-PopZ focus also localized at the stalked pole. In cells with CFP-ParB localized at the new pole, ∼90% displayed PopZ-CFP foci located at each pole. Notably, cells with CFP-ParB localized at mid-cell displayed an equal combination of cells with either one mCherry-PopZ focus localized at the stalked pole or mCherry-PopZ foci localized at each pole. Upon the induction of chromosome replication by the addition of the inducer vanillate, cells with bipolar localization of PopZ remained bipolar (Fig. 7B). Regardless of where the centromere was localized when replication initiation was induced by vanillate supplementation, ∼100% of cells displayed bipolar localization of mCherry-PopZ subsequent to the onset of chromosome replication. Our data suggest that the initiation of chromosome replication and segregation influence the bipolar localization of PopZ.

### Effects in viability from initiating chromosome replication/segregation from outside the stalked pole.

**FIG 8:**
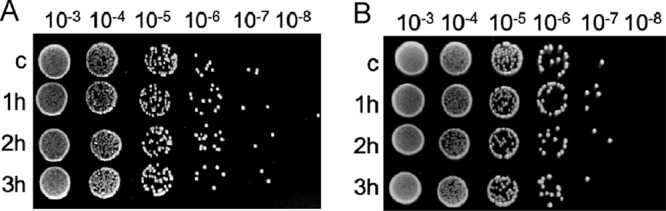
Depletion of DnaA for 3 h does not alter the viability of *Caulobacter*. (A and B) CFU assays of PM500 cells [*parS*(pMT1) *vanA*::*dnaA* Δ*dnaA xylX*::*cfp*-*parB*(pMT1)] (A) and PM109 cells (Δ*vanA parB*::*cfp*-*parB dnaA*::Ω *vanA*::*dnaA*) (B). The cultures (3 ml) grown to an OD_600_ of ∼0.3 were washed three times with 1× M2 salts as described in Materials and Methods, and the OD_600_ was set at ∼ 0.2 in M2G medium (2 ml). DnaA was depleted for 1, 2, and 3 h in separate cultures at 28°C, and the CFU assay was performed. Control sample (c) were not depleted of DnaA and were incubated with vanillate (250 μM) for 3 h. PYE plates supplemented with vanillate (250 μM) were incubated at 28°C for 2 days prior to obtaining the images. The data shown are representative of three independent replicates.

To determine whether initiating chromosome replication and/or segregation from outside the stalked pole altered the viability of *Caulobacter*, we analyzed CFU of cells that had undergone *ori* or centromere translocation away from the stalked pole in the absence of replication (Fig. 8). Cells of strains PM500 (fluorescent label near *ori*) and PM109 (fluorescently labeled ParB-centromere) were spotted immediately after DnaA depletion (1 h to 3 h) on plates containing the inducer for *dnaA* expression (supplemented with vanillate). Our data revealed no significant differences between cells that initiated chromosome replication and segregation from the new pole (or mid-cell) compared to wild-type conditions. Our data suggest that cells can recover relatively quickly after initiating chromosome replication/segregation from outside the stalked pole.

## DISCUSSION

By inducing the movement of *ori* and centromere away from their intrinsic subcellular locations in a replication-independent manner, we have shown that the molecular machinery involved in chromosome replication and segregation are remarkably flexible with respect to their subcellular orientation. We found that the activity of DnaA and ParA are not restricted to a single polar microdomain and can successfully induce chromosome replication and segregation from the opposite polar microdomain composed of distinct client proteins. These results suggest that the chromosomal loci *ori* and *parS* play key roles in the localization of cell cycle regulators. Cells that initiate chromosome replication and segregation from outside the stalked pole displayed no detectable viability defects compared to wild-type conditions. Therefore, our data reveal the ability of the cell to rapidly reorganize chromosome orientation along with the set of proteins involved in chromosomal replication and segregation.

### DnaA’s activity as replication initiator and cell asymmetry.

In the absence of membrane-bound organelles, bacteria rely on proteins organized in gradients to establish cellular polarity and perform asymmetric functions. One example of such organization is the phosphorylated regulator CtrA (CtrA∼P), which binds *ori* and inhibits DnaA from initiating replication at one cell pole (23–25). In predivisional cells, a phospho-signaling relay at the cell poles has been proposed to generate an asymmetric concentration gradient of CtrA∼P with the highest levels at the new pole that gradually decrease toward the stalked pole (26). Alterations to this proposed asymmetric concentration gradient of CtrA∼P eliminate the asymmetric regulation of DnaA, resulting in cells initiating replication from the new pole (26). Our data revealed that DnaA can also trigger replication initiation from the new pole in cells with altered subcellular location of *ori* (Fig. 3). Our results can be explained by the undetected levels of CtrA in cells depleted of DnaA (27, 28). This is because DnaA is a transcriptional regulator of *gcrA*, and GcrA is a transcriptional regulator of *ctrA* (27–29). Consequently, the expression of *ctrA* is indirectly dependent on the levels of DnaA. Thus, cells with *ori* localized at the new pole with depleted levels of DnaA are likely to have no CtrA∼P or a minimal CtrA∼P gradient that is insufficient to inhibit replication initiation from either pole.

### Regulation of periodicity of DnaA activity.

Most of the regulators of DnaA activity that have been identified thus far are negative regulators that prevent the overinitiation of chromosome replication. However, positive regulators that trigger DnaA to initiate replication with such efficient periodicity remain limited. This periodicity of DnaA activity is maintained even in E. coli cells that were artificially designed to have two spatially separated *ori* foci. In those cells, DnaA productively initiated replication synchronously from both *ori* foci (30). In E. coli and Helicobacter pylori, a recruiter of DnaA has been characterized that promotes the assembly of polymeric DnaA at *ori* (31–33). Notably, constitutive expression of *dnaA* in *Caulobacter* has been shown to have no effect on the periodicity of DnaA activity, suggesting that *dnaA* transcriptional regulation is not the principal modulator of DnaA periodicity (34). In *Caulobacter*, CtrA regulates the spatial activity of DnaA so that chromosome replication initiates only in the stalked cells (24, 25, 34). However, CtrA is not involved in the periodicity of DnaA’s activity (34). Thus, the molecular mechanism that triggers DnaA to initiate chromosome replication with such precise periodicity remains unclear. A hypothetical scenario is that some type of regulator that facilitates this periodicity process is found within the microenvironment at *ori*’s location at the time of replication initiation. Our data suggest that there is no regulator/modulator of DnaA that is fixed at the stalked pole’s microdomain. We cannot however exclude the possibility that a potential modulator does exist and that this modulator could migrate along with *ori* because either it binds directly to *ori* or it is recruited by DnaA. Biochemical characterization of DnaA’s activity at *ori* is required to identify the mechanism that DnaA uses to regulate its temporal activity with such remarkable accuracy.

### Localization of the centromere dictates the orientation of ParA’s activity.

The partitioning protein ParA is another example of a bacterial protein organized in a gradient to establish cellular polarity. In *Caulobacter*, ParA forms a gradient with concentrations gradually decreasing from the new pole to the stalked pole (10–12, 20). Interestingly, this stable gradient of ParA is established well before chromosome replication and segregation are initiated (10–12). Thus, the question remains as to what activates this asymmetric organization of ParA. Our data suggest that the organization of the ParA gradient can be reconstructed in the opposite orientation by rearranging the location of the *parS*-ParB complex (Fig. 6). Cells with *parS*-ParB at the new pole displayed higher levels of ParA at the stalked pole than at the new pole.

We propose that the localization of the centromere complex directs the arrangement of the ParA gradient. This model is consistent with how ParA partitioning systems segregate low-copy-number plasmids to maintain inheritance. Using *in vitro* reconstitution assays, the ParB-*parS* complexes of these plasmids were shown to chase and rearrange the ParA gradient (35–37). For chromosome segregation, the specific component of the centromere complex that triggers the organization of ParA remains to be determined. There are several proteins that bind *parS* and/or interact with *parS*-ParB that could serve as potential regulators of ParA’s subcellular organization. One possibility is that ParB itself triggers ParA’s organization by inducing ParA to hydrolyze ATP (9, 10, 14), consistent with what has been observed with partitioning of plasmids (35–37). Another possibility is MipZ, the inhibitor of FtsZ polymerization, which colocalizes with the *parS*-ParB complex (38). The replication initiator DnaA is another candidate because it also binds *parS* and has been proposed to be involved in the onset of centromere segregation (16). Last, the anchoring protein PopZ, which interacts with ParB, has been proposed to be involved in ParA activity and directionality (6–8, 19, 22). Our data suggest that the bipolar localization of PopZ is elicited primarily by centromere segregation, as suggested previously (39). About 90% of cells displayed PopZ bipolarly localized in cells with an unreplicated centromere translocated to the new pole (Fig. 7). However, only upon the onset of chromosome replication did we observe 100% of cells with bipolarly localized PopZ, suggesting that replication initiation may also influence PopZ localization. It remains to be determined whether PopZ plays a role in the organization of ParA.

We have demonstrated that ParA can successfully segregate the *parS* centromere from the new pole to the stalked pole, which is the reverse direction to that observed in wild-type cells. We propose that once a stable gradient of ParA is formed in cells with translocated unreplicated centromeres, the ParA-DNA interaction relay previously shown to provide the force necessary for centromere segregation (20) can initiate and segregate one centromere in the reverse direction.

### Robustness of cells to reorganize.

Establishment of cellular polarity is required for asymmetric cell division. Notably, the signaling factors involved in establishing polarity in C. crescentus are conserved among bacteria from diverse environmental niches (22, 40–44), like Brucella abortus (causative agent for brucellosis in mammals) (45, 46), Sinorhizobium meliloti (plant symbiont) (47), and Agrobacterium tumefaciens (plant pathogen) (48). However, little is known about how the gradients of these signaling polarity factors are formed or how they function in bacteria with diverse life styles. In this work, we asked what happens to the ability of cells to grow when the organization of the two highly conserved chromosomal loci (*ori* and *parS* centromere) are flipped in orientation. We showed that the regulators (DnaA and ParA) can easily adapt to the new locations of these sites and proceed with their activities, and in the case of ParA proceed to orient segregation in the reverse direction. Remarkably, cells were able to recover the “forced” rearrangement of these chromosomal loci and continue to grow with no measurable delays. Our results revealed the robustness and flexibility that cells have to rearrange their signaling polarity factors.

## MATERIALS AND METHODS

### Bacterial strains and growth conditions.

Lists of strains, plasmids, and primers used in this work are provided in Tables S1 and S2 in the supplemental material. Plasmids constructed in this study were created by cloning PCR products amplified using wild-type CB15N (NA1000) or Yersinia pestis KIM5 pMT1 genomic DNA into pNPTS138, pXCHYC-2, or pXCFPN-2 vectors (49). The constructs were transformed into E. coli DH5α cells and grown at 37°C in Luria-Bertani (LB) medium. All primers used for cloning are listed in Table S2. Plasmid carrying *cfp*-*parB*(pMT1) was done by the *parB*(pMT1) gene sequence isolation with KpnI and NheI restriction from PM396 (LS5269) and ligation to the equally treated xylose-inducible integrating plasmid pXCFPN-2 (Kan^r^) (9). The *parA* gene was cloned into integrating pXCHYC-2 (Kan^r^) plasmid under a xylose-inducible promoter to express mCherry-tagged C-terminal protein fusions (49). The Gibson cloning method (50) was used to construct the plasmids used to delete or insert a gene into the *Caulobacter* genome.

10.1128/mBio.01002-19.4 TABLE S1 List of strains and plasmids used in this study. Download Table S1, DOCX file, 0.03 MB. Copyright © 2019 Meléndez et al. 2019 Meléndez et al. This content is distributed under the terms of the Creative Commons Attribution 4.0 International license.

10.1128/mBio.01002-19.5 TABLE S2 List of primers used in this study. Primers that start with “Gib” were used in Gibson cloning technique. Download Table S2, DOCX file, 0.02 MB. Copyright © 2019 Meléndez et al. 2019 Meléndez et al. This content is distributed under the terms of the Creative Commons Attribution 4.0 International license.

### Construction of indicator strain PM500 with fluorescently labeled origin of replication.

To track the cellular localization of *ori*, we engineered a fluorescent tag to be inserted near *ori* using the Y. pestis *parS*(pMT1) chromosomal sequence and its corresponding gene encoding ParB(pMT1) (17). To insert the *parS*(pMT1) site approximately 1 kb far away from the *ori*, the cloned *parS*(pMT1) sequence from PM395 (LS5270) and around 600 bp of CCNA0001 C-terminal and CCNA0002 N-terminal sequences were assembled into the pNPTS138 plasmid (9). The *parS*-ParB(pMT1) system from *Yersinia* has been previously used in *Caulobacter* and shown not to interfere with the activity of the native *Caulobacter* ParABS partitioning system (9). To control the expression of *dnaA*, we first engineered an additional copy of *dnaA* to replace the *vanA* gene, resulting in the expression of *dnaA* regulated by the VanA promoter (*vanA*::*dnaA*) (49). Using this merodiploid strain, the native gene encoding DnaA was deleted, leaving no scars on the genome. The final indicator strain PM500 has the genotype *xylX*::*cfp*-*parB*(pMT1) *parS*(pMT1) at nucleotide 1108, *dnaA*::Ω, *vanA*::*dnaA*. We refer here to the *parS*(pMT1) localized near *ori* simply as *ori*.

### Growth assays.

Overnight cultures grown from *Caulobacter* frozen stocks in M2G liquid medium were diluted to an optical density at 600 nm (OD_600_) of 0.2 (2 ml) in 13-mm glass tubes. Cultures were incubated at 28°C, and the optical density at 600 nm was monitored every hour to monitor the growth rates of bacteria.

### Synchronization.

A culture of *Caulobacter* in M2G (15 ml) was inoculated with a saturated overnight M2G culture and grown to an OD_600_ of ∼0.3. The medium was supplemented with vanillate (250 μM) and antibiotics as noted. Cells were pelleted using centrifugation at 6,000 rpm for 10 min at 4°C. The cell pellet was resuspended in about 800 μl of 1× M2 salts and mixed well with Percoll (900 μl; Sigma-Aldrich) to generate a density gradient. Swarmer cells (bottom layer) were separated out from the stalked/predivisional cells (top layer) by centrifuging at 11,000 rpm for 20 min at 4°C. Collected swarmer cells were washed twice with cold 1× M2 salts by spinning at 8,000 rpm for 3 min at 4°C and resuspended in M2G medium to the appropriate OD_600_. When cells were not synchronized, the cultures grown to an OD_600_ of ∼0.3 were pelleted and washed with 1× M2 salts three times.

### Fluorescence microscopy.

The cells (1 to 3 μl) were spotted on agar pads (1% agarose in M2G) and imaged using phase-contrast and fluorescence microscopy in Zeiss Axio Observer 2.1 inverted microscope, set up with a Plan-Apochromat 100×/1.40 Oil Ph3 M27 (WD = 0.17 mm) objective, AxioCam 506 mono camera and ZEN lite software. Agar pads supplemented with vanillate (250 μM) were used in time-lapse assays when needed. Images were analyzed using Fiji software (51), and localization of fluorescent foci was counted using the Cell Counter plugin.

### CFU assay.

The cultures were serially diluted (10-fold) by mixing 10 μl of culture with 90 μl of PYE medium in a sterile 96-well plate. Five microliters of each sample was spotted onto PYE agar (1.5%) plates supplemented with vanillate (250 μM) if needed. CFU counts were obtained from the plates incubated at 28°C for 2 days.

## References

[1] Hwang DS, Kornberg A 1992 Opening of the replication origin of *Escherichia coli* by DnaA protein with protein HU or IHF. J Biol Chem 267:23083–23086. 1429655

[2] Boye E, Lobner-Olesen A, Skarstad K 2000 Limiting DNA replication to once and only once. EMBO Rep 1:479–483. doi:10.1093/embo-reports/kvd116. 11263490 PMC1083788

[3] Sekimizu K, Bramhill D, Kornberg A 1988 Sequential early stages in the in vitro initiation of replication at the origin of the Escherichia coli chromosome. J Biol Chem 263:7124–7130. 2835363

[4] Katayama T, Ozaki S, Keyamura K, Fujimitsu K 2010 Regulation of the replication cycle: conserved and diverse regulatory systems for DnaA and oriC. Nat Rev Microbiol 8:163–170. doi:10.1038/nrmicro2314. 20157337

[5] Erzberger JP, Mott ML, Berger JM 2006 Structural basis for ATP-dependent DnaA assembly and replication-origin remodeling. Nat Struct Mol Biol 13:676–683. doi:10.1038/nsmb1115. 16829961

[6] Bowman GR, Comolli LR, Gaietta GM, Fero M, Hong SH, Jones Y, Lee JH, Downing KH, Ellisman MH, McAdams HH, Shapiro L 2010 Caulobacter PopZ forms a polar subdomain dictating sequential changes in pole composition and function. Mol Microbiol doi:10.1111/j.1365-2958.2010.07088.x. PMC2935252 20149103

[7] Bowman GR, Comolli LR, Zhu J, Eckart M, Koenig M, Downing KH, Moerner WE, Earnest T, Shapiro L 2008 A polymeric protein anchors the chromosomal origin/ParB complex at a bacterial cell pole. Cell 134:945–955. doi:10.1016/j.cell.2008.07.015. 18805088 PMC2745220

[8] Ebersbach G, Briegel A, Jensen GJ, Jacobs-Wagner C 2008 A self-associating protein critical for chromosome attachment, division, and polar organization in caulobacter. Cell 134:956–968. doi:10.1016/j.cell.2008.07.016. 18805089 PMC2614312

[9] Toro E, Hong SH, McAdams HH, Shapiro L 2008 Caulobacter requires a dedicated mechanism to initiate chromosome segregation. Proc Natl Acad Sci U S A 105:15435–15440. doi:10.1073/pnas.0807448105. 18824683 PMC2563096

[10] Ptacin JL, Lee SF, Garner EC, Toro E, Eckart M, Comolli LR, Moerner WE, Shapiro L 2010 A spindle-like apparatus guides bacterial chromosome segregation. Nat Cell Biol 12:791–798. doi:10.1038/ncb2083. 20657594 PMC3205914

[11] Schofield WB, Lim HC, Jacobs-Wagner C 2010 Cell cycle coordination and regulation of bacterial chromosome segregation dynamics by polarly localized proteins. EMBO J 29:3068–3081. doi:10.1038/emboj.2010.207. 20802464 PMC2944072

[12] Shebelut CW, Guberman JM, van Teeffelen S, Yakhnina AA, Gitai Z 2010 Caulobacter chromosome segregation is an ordered multistep process. Proc Natl Acad Sci U S A 107:14194–14198. doi:10.1073/pnas.1005274107. 20660743 PMC2922572

[13] Viollier PH, Thanbichler M, McGrath PT, West L, Meewan M, McAdams HH, Shapiro L 2004 Rapid and sequential movement of individual chromosomal loci to specific subcellular locations during bacterial DNA replication. Proc Natl Acad Sci U S A 101:9257–9262. doi:10.1073/pnas.0402606101. 15178755 PMC438963

[14] Easter J Jr, Gober JW 2002 ParB-stimulated nucleotide exchange regulates a switch in functionally distinct ParA activities. Mol Cell 10:427–434. doi:10.1016/S1097-2765(02)00594-4. 12191487

[15] Surovtsev IV, Lim HC, Jacobs-Wagner C 2016 The slow mobility of the ParA partitioning protein underlies its steady-state patterning in Caulobacter. Biophys J 110:2790–2799. doi:10.1016/j.bpj.2016.05.014. 27332137 PMC4919595

[16] Mera PE, Kalogeraki VS, Shapiro L 2014 Replication initiator DnaA binds at the Caulobacter centromere and enables chromosome segregation. Proc Natl Acad Sci U S A 111:16100–16105. doi:10.1073/pnas.1418989111. 25349407 PMC4234595

[17] Lindler LE, Plano GV, Burland V, Mayhew GF, Blattner FR 1998 Complete DNA sequence and detailed analysis of the Yersinia pestis KIM5 plasmid encoding murine toxin and capsular antigen. Infect Immun 66:5731–5742. 9826348 10.1128/iai.66.12.5731-5742.1998 PMC108724

[18] Mohl DA, Gober JW 1997 Cell cycle-dependent polar localization of chromosome partitioning proteins in Caulobacter crescentus. Cell 88:675–684. doi:10.1016/s0092-8674(00)81910-8. 9054507

[19] Ptacin JL, Gahlmann A, Bowman GR, Perez AM, von Diezmann AR, Eckart MR, Moerner WE, Shapiro L 2014 Bacterial scaffold directs pole-specific centromere segregation. Proc Natl Acad Sci U S A 111:E2046–E2055. doi:10.1073/pnas.1405188111. 24778223 PMC4024888

[20] Lim HC, Surovtsev IV, Beltran BG, Huang F, Bewersdorf J, Jacobs-Wagner C 2014 Evidence for a DNA-relay mechanism in ParABS-mediated chromosome segregation. Elife 3:e02758. doi:10.7554/eLife.02758. 24859756 PMC4067530

[21] Murray H, Errington J 2008 Dynamic control of the DNA replication initiation protein DnaA by Soj/ParA. Cell 135:74–84. doi:10.1016/j.cell.2008.07.044. 18854156

[22] Berge M, Viollier PH 2018 End-in-sight: cell polarization by the polygamic organizer PopZ. Trends Microbiol 26:363–375. doi:10.1016/j.tim.2017.11.007. 29198650

[23] Laub MT, Chen SL, Shapiro L, McAdams HH 2002 Genes directly controlled by CtrA, a master regulator of the Caulobacter cell cycle. Proc Natl Acad Sci U S A 99:4632–4637. doi:10.1073/pnas.062065699. 11930012 PMC123699

[24] Quon KC, Marczynski GT, Shapiro L 1996 Cell cycle control by an essential bacterial two-component signal transduction protein. Cell 84:83–93. doi:10.1016/s0092-8674(00)80995-2. 8548829

[25] Quon KC, Yang B, Domian IJ, Shapiro L, Marczynski GT 1998 Negative control of bacterial DNA replication by a cell cycle regulatory protein that binds at the chromosome origin. Proc Natl Acad Sci U S A 95:120–125. doi:10.1073/pnas.95.1.120. 9419339 PMC18146

[26] Chen YE, Tropini C, Jonas K, Tsokos CG, Huang KC, Laub MT 2011 Spatial gradient of protein phosphorylation underlies replicative asymmetry in a bacterium. Proc Natl Acad Sci U S A 108:1052–1057. doi:10.1073/pnas.1015397108. 21191097 PMC3024676

[27] Hottes AK, Shapiro L, McAdams HH 2005 DnaA coordinates replication initiation and cell cycle transcription in Caulobacter crescentus. Mol Microbiol 58:1340–1353. doi:10.1111/j.1365-2958.2005.04912.x. 16313620

[28] Collier J, Murray SR, Shapiro L 2006 DnaA couples DNA replication and the expression of two cell cycle master regulators. EMBO J 25:346–356. doi:10.1038/sj.emboj.7600927. 16395331 PMC1383511

[29] Laub MT, McAdams HH, Feldblyum T, Fraser CM, Shapiro L 2000 Global analysis of the genetic network controlling a bacterial cell cycle. Science 290:2144–2148. doi:10.1126/science.290.5499.2144. 11118148

[30] Wang X, Lesterlin C, Reyes-Lamothe R, Ball G, Sherratt DJ 2011 Replication and segregation of an Escherichia coli chromosome with two replication origins. Proc Natl Acad Sci U S A 108:E243–E250. doi:10.1073/pnas.1100874108. 21670292 PMC3127894

[31] Zawilak-Pawlik A, Donczew R, Szafrański S, Mackiewicz P, Terradot L, Zakrzewska-Czerwińska J 2011 DiaA/HobA and DnaA: a pair of proteins co-evolved to cooperate during bacterial orisome assembly. J Mol Biol 408:238–251. doi:10.1016/j.jmb.2011.02.045. 21354425

[32] Ishida T, Akimitsu N, Kashioka T, Hatano M, Kubota T, Ogata Y, Sekimizu K, Katayama T 2004 DiaA, a novel DnaA-binding protein, ensures the timely initiation of Escherichia coli chromosome replication. J Biol Chem 279:45546–45555. doi:10.1074/jbc.M402762200. 15326179

[33] Zawilak-Pawlik A, Kois A, Stingl K, Boneca IG, Skrobuk P, Piotr J, Lurz R, Zakrzewska-Czerwińska J, Labigne A 2007 HobA–a novel protein involved in initiation of chromosomal replication in Helicobacter pylori. Mol Microbiol 65:979–994. doi:10.1111/j.1365-2958.2007.05853.x. 17645450

[34] Jonas K, Chen YE, Laub MT 2011 Modularity of the bacterial cell cycle enables independent spatial and temporal control of DNA replication. Curr Biol 21:1092–1101. doi:10.1016/j.cub.2011.05.040. 21683595 PMC3143580

[35] Vecchiarelli AG, Hwang LC, Mizuuchi K 2013 Cell-free study of F plasmid partition provides evidence for cargo transport by a diffusion-ratchet mechanism. Proc Natl Acad Sci U S A 110:E1390–E1397. doi:10.1073/pnas.1302745110. 23479605 PMC3625265

[36] Hwang LC, Vecchiarelli AG, Han YW, Mizuuchi M, Harada Y, Funnell BE, Mizuuchi K 2013 ParA-mediated plasmid partition driven by protein pattern self-organization. EMBO J 32:1238–1249. doi:10.1038/emboj.2013.34. 23443047 PMC3642677

[37] Vecchiarelli AG, Neuman KC, Mizuuchi K 2014 A propagating ATPase gradient drives transport of surface-confined cellular cargo. Proc Natl Acad Sci U S A 111:4880–4885. doi:10.1073/pnas.1401025111. 24567408 PMC3977271

[38] Thanbichler M, Shapiro L 2006 MipZ, a spatial regulator coordinating chromosome segregation with cell division in Caulobacter. Cell 126:147–162. doi:10.1016/j.cell.2006.05.038. 16839883

[39] Laloux G, Jacobs-Wagner C 2013 Spatiotemporal control of PopZ localization through cell cycle-coupled multimerization. J Cell Biol 201:827–841. doi:10.1083/jcb.201303036. 23751494 PMC3678156

[40] Collier J 2016 Cell cycle control in Alphaproteobacteria. Curr Opin Microbiol 30:107–113. doi:10.1016/j.mib.2016.01.010. 26871482

[41] Panis G, Murray SR, Viollier PH 2015 Versatility of global transcriptional regulators in alpha-Proteobacteria: from essential cell cycle control to ancillary functions. FEMS Microbiol Rev 39:120–133. doi:10.1093/femsre/fuu002. 25793963

[42] Mohapatra SS, Fioravanti A, Biondi EG 2014 DNA methylation in Caulobacter and other Alphaproteobacteria during cell cycle progression. Trends Microbiol 22:528–535. doi:10.1016/j.tim.2014.05.003. 24894626

[43] Wang H, Ziesche L, Frank O, Michael V, Martin M, Petersen J, Schulz S, Wagner-Döbler I, Tomasch J 2014 The CtrA phosphorelay integrates differentiation and communication in the marine alphaproteobacterium Dinoroseobacter shibae. BMC Genomics 15:130. doi:10.1186/1471-2164-15-130. 24524855 PMC4046655

[44] Poncin K, Gillet S, De Bolle X 2018 Learning from the master: targets and functions of the CtrA response regulator in Brucella abortus and other alpha-proteobacteria. FEMS Microbiol Rev 42:500–513. doi:10.1093/femsre/fuy019. 29733367

[45] Hallez R, Mignolet J, Van Mullem V, Wery M, Vandenhaute J, Letesson JJ, Jacobs-Wagner C, De Bolle X 2007 The asymmetric distribution of the essential histidine kinase PdhS indicates a differentiation event in Brucella abortus. EMBO J 26:1444–1455. doi:10.1038/sj.emboj.7601577. 17304218 PMC1817626

[46] Deghelt M, Mullier C, Sternon JF, Francis N, Laloux G, Dotreppe D, Van der Henst C, Jacobs-Wagner C, Letesson JJ, De Bolle X 2014 G1-arrested newborn cells are the predominant infectious form of the pathogen Brucella abortus. Nat Commun 5:4366. doi:10.1038/ncomms5366. 25006695 PMC4104442

[47] Pini F, De Nisco NJ, Ferri L, Penterman J, Fioravanti A, Brilli M, Mengoni A, Bazzicalupo M, Viollier PH, Walker GC, Biondi EG 2015 Cell cycle control by the master regulator CtrA in Sinorhizobium meliloti. PLoS Genet 11:e1005232. doi:10.1371/journal.pgen.1005232. 25978424 PMC4433202

[48] Ehrle HM, Guidry JT, Iacovetto R, Salisbury AK, Sandidge DJ, Bowman GR 2017 Polar organizing protein PopZ is required for chromosome segregation in Agrobacterium tumefaciens. J Bacteriol 199:e00111-17. doi:10.1128/JB.00111-17. 28630129 PMC5553026

[49] Thanbichler M, Iniesta AA, Shapiro L 2007 A comprehensive set of plasmids for vanillate- and xylose-inducible gene expression in Caulobacter crescentus. Nucleic Acids Res 35:e137. doi:10.1093/nar/gkm818. 17959646 PMC2175322

[50] Gibson DG, Young L, Chuang RY, Venter JC, Hutchison CA III, Smith HO 2009 Enzymatic assembly of DNA molecules up to several hundred kilobases. Nat Methods 6:343–345. doi:10.1038/nmeth.1318. 19363495

[51] Schindelin J, Arganda-Carreras I, Frise E, Kaynig V, Longair M, Pietzsch T, Preibisch S, Rueden C, Saalfeld S, Schmid B, Tinevez J-Y, White DJ, Hartenstein V, Eliceiri K, Tomancak P, Cardona A 2012 Fiji: an open-source platform for biological-image analysis. Nat Methods 9:676. doi:10.1038/nmeth.2019. 22743772 PMC3855844

