# Supplementary figures and images for: Chromosome Dynamics in Bacteria: Triggering Replication at the Opposite Location and Segregation in the Opposite Direction
Source: mBio. 2019 Jul 30;10(4):e01002-19. doi: 10.1128/mBio.01002-19 (PMC6667618; doi:10.1128/mBio.01002-19)

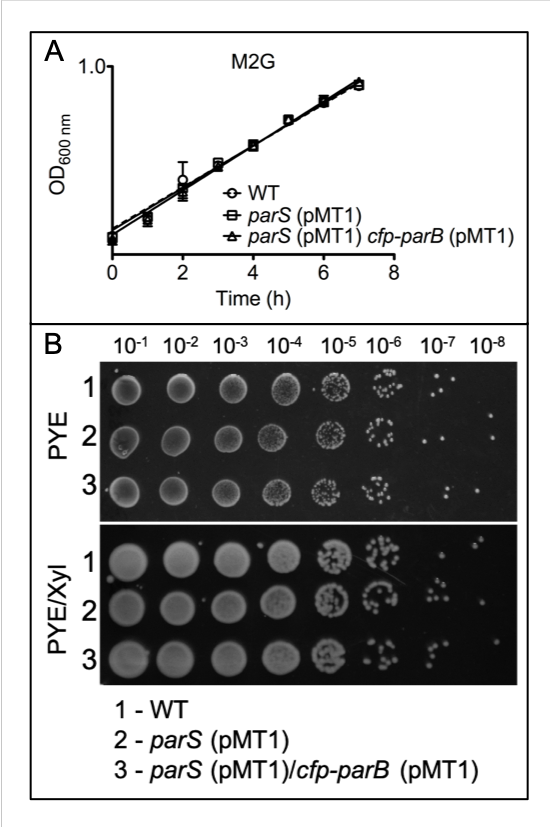

Supplement: FIG S1 [file mBio.01002-19-sf001.tif]

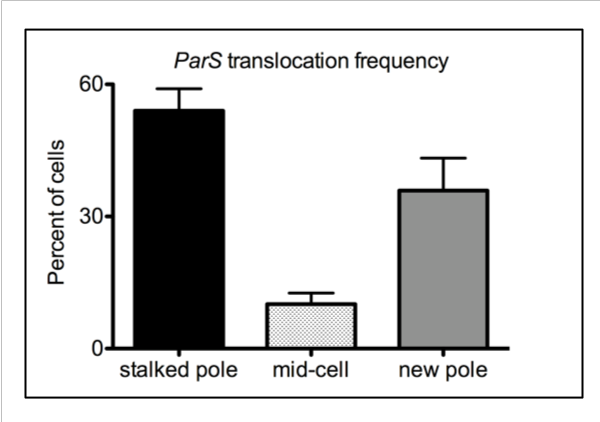

Supplement: FIG S2 [file mBio.01002-19-sf002.tif]

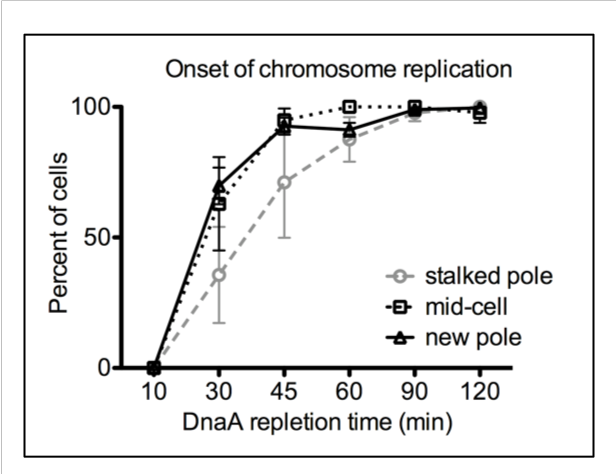

Supplement: FIG S3 [file mBio.01002-19-sf003.tif]
