# Supplementary material for: Chromosome Dynamics in Bacteria: Triggering Replication at the Opposite Location and Segregation in the Opposite Direction
Source: mBio. 2019 Jul 30;10(4):e01002-19. doi: 10.1128/mBio.01002-19 (PMC6667618; doi:10.1128/mBio.01002-19)
Supplement: TABLE S1 [file mBio.01002-19-st001.docx]

| **Strain or plasmid** | **Description** | **Source** |
| --- | --- | --- |
| ***Caulobacter* strains** |  |  |
| PM1 | Wild-type CB15N (NA1000) | (1) |
| PM109 | CB15N, Δ*vanA*, *parB*∷*cfp*-*parB,* *dnaA*::Ω (strp^R^/spec^R^), *vanA*::*dnaA* | (2) |
| PM121 | *xylX*::*parA(D44A)-yfp* (tet^R^) in PM109 (strp^R^) | This study |
| PM247 | *xylX*::*mCherry-PopZ* (tet^R^) in PM109 (strp^R^) | (2) |
| PM433 | CB15N, pMT1 *parS* | This study |
| PM438 | CB15N, pMT1 *parS,* xylX::*yfp*-*parB*(pMT1) (kan^R^) | This study |
| PM500 | CB15N *parS* (pMT1), *vanA::dnaA,* Δ*dnaA, xylX*::*cfp*-*parB*(pMT1) (kan^R^) | This study |
| PM503 | pDNA219 (*parA-mCherry* under xylose promoter) (kan^R^) in PM109 (strp^R^) | This study |
|  |  |  |
| **Plasmids** |  |  |
| pNPTS138 | Nonreplicating vector for allelic replacement (kan^R^) *oriT* *sacB* | Alley M. R. K., unpublished data |
| pXCHYC-2 | Integrating plasmid for xylose inducible expression of mCherry tagged CCNA_03869 (*parA)*; (kan^R^) | (3) |
| pXCFPN-2 | Integrating plasmid for xylose inducible expression of CFP tagged *parB(pMT1)* (kan^R^) | (3) |
| pDNA214 | pNPTS138- *parS* (pMT1) sequence flanked by 600bp UP/DWN at base 1108 for insertion of *parS*(pMT1) near *ori*. | This study |
| pDNA216 | pXCFPN-2 – *xylX*::*cfp*-*parB* (pMT1) (kan^R^) | This study |
| pDNA217 | pNPTS138- *dnaA* sequence flanked by 600bp UP/DWN of CCNA_02476. To replace *vanA* with *dnaA* | This study |
| pDNA218 | pNPTS138- 600bp UP/DWN CCNA_00008 for *dnaA* deletion | This study |
| pJP49 | pXYFPC5-*parA*(D44A) | (4) |
| pDNA219 | pXCHYC-2- CCNA_03869 cloned at NdeI 5' and SacI 3' restriction enzyme sites | This study |
| pMS138 | pMCS4 – *parS*(pMT1) | (5) |
| pMS139 | pXYFPN-2 - *xylX* ::y*fp*-*parB*( pMT1) (kan^R^) | (5) |

**References**

1. Evinger M, Agabian N. 1979. Caulobacter crescentus nucleoid: analysis of sedimentation behavior and protein composition during the cell cycle. Proceedings of the National Academy of Sciences of the United States of America 76:175-178.

2. Mera PE, Kalogeraki VS, Shapiro L. 2014. Replication initiator DnaA binds at the Caulobacter centromere and enables chromosome segregation. Proc Natl Acad Sci U S A 111:16100-5.

3. Thanbichler M, Iniesta AA, Shapiro L. 2007. A comprehensive set of plasmids for vanillate- and xylose-inducible gene expression in Caulobacter crescentus. Nucleic acids research 35:e137-e137.

4. Ptacin JL, Lee SF, Garner EC, Toro E, Eckart M, Comolli LR, Moerner WE, Shapiro L. 2010. A spindle-like apparatus guides bacterial chromosome segregation. Nature Cell Biology 12:791.

5. Schwartz MA, Shapiro L. 2011. An SMC ATPase mutant disrupts chromosome segregation in Caulobacter. Molecular microbiology 82:1359-1374.
