# Supplementary material for: Chromosome Dynamics in Bacteria: Triggering Replication at the Opposite Location and Segregation in the Opposite Direction
Source: mBio. 2019 Jul 30;10(4):e01002-19. doi: 10.1128/mBio.01002-19 (PMC6667618; doi:10.1128/mBio.01002-19)
Supplement: TABLE S2 [file mBio.01002-19-st002.docx]

| **Primer name** | **5’ to 3’ sequence** |
| --- | --- |
| Gib_UP600dnaA_fow | AGCTTCTCTGCAGGATATCTGGATCCGCGCGTGCGCACCTTC |
| Gib_UP600dnaA_rev | TCTTCAATCCTACGATACGGTTTCGTCGTCCACCGCCTTGCA |
| Gib_DWN600dnaA_fow | TGTTTTTGTGCAAGGCGGTGGACGACGAAACCGTATCGTAGG |
| Gib_DWN600dnaA_rev | GCCGAAGCTAGCGAATTCGTGGATCTCGAAGCGGTGCTAGCG |
| Gib_UP600bpVanA_fow | AGCTTCTCTGCAGGATATCTGGATCGGGCGCTCTCGACAGCG |
| Gib_UP600vanA_rev | TGGCAACCCCGCCCTTCATGGTCATCGTCGTTTCCTCGCATC |
| Gib_dnaA_fow | CCGAACCACGATGCGAGGAAACGACGATGACCATGAAGGGCGG |
| Gib_dnaA_rev | GCGCGGGACGCCACCCGAACCTTGATTAGCCCCGCAGCTTGC |
| Gib_DWN600vanA_fow | CCTGACGCGCAAGCTGCGGGGCTAATCAAGGTTCGGGTGGCG |
| Gib_DWN600vanA_rev | GCCGAAGCTAGCGAATTCGTGGATCCGTCGATGACGTGGGTC |
| Gib_CCNA_parSpMT1Up-fwd | GCTTCTCTGCAGGATATCTGGATCGGCTGCCGCAAGCTGGA |
| S_Gib_CCNA_parSpMT1Up-rev | TGAAATCACCACGCTTTTCAACCTCTCATCCCAGGACCTCGA |
| S_Gib_ParSpMT1_fow | GGCAAGGTCGAGGTCCTGGGATGAGAGGTTGAAAAGCGTGG |
| S_Gib_ParSpMT1_rev | CGAGCGTAACAGGGGTGAGGCTCATAAGCTTTGTTTTTCACC |
| S_Gib_CCNA_parSpMT1DWN_fwd | GAAATTGGCGTGGTGAAAAACAAAGCTTATGAGCCTCACCCCTG |
| Gib_CCNA_parSpMT1DWN_rev | CGAAGCTAGCGAATTCGTGGATCTCATACGGTCAGGGCTC |
| CCNA_ParA atg_5'NdheI_fow | AAACATATGTCCGCTAATCCTCTCCG |
| CCNA_ParA -3'-SacI_rev | AAAGAGCTCTGGCGGCCTTGGCCTG |
